# Targeting educational campaigns for prevention of malaria and dengue fever: an assessment in Thailand

**DOI:** 10.1186/s13071-015-0653-4

**Published:** 2015-01-23

**Authors:** Macy Brusich, John Grieco, Naomi Penney, Rungarun Tisgratog, Wanapa Ritthison, Theeraphap Chareonviriyaphap, Nicole Achee

**Affiliations:** Eck Institute for Global Health, University of Notre Dame, 120 Brownson Hall, Notre Dame, IN 46556 USA; Department of Entomology, Faculty of Agriculture, Kasetsart University, 50 Ngam Wong Wan Road, Chatuchak, Bangkok, 10900 Thailand; The Office of Disease Prevention and Control 3 Chonburi, Vachiraprakarn Road, Bansuan, Subdistrict, MuangDstrict, Chonburi, 20000 Thailand

**Keywords:** Malaria, Dengue fever, Education campaigns, Vector-control, Knowledge, Attitudes, and Practices (KAP), Thailand

## Abstract

**Background:**

The current study assessed the knowledge, attitudes, and practices (KAP) of at-risk populations for malaria and/or dengue fever in relation to mosquito exposure and household mosquito control practices. Specific objectives included comparison of individual and household level health practices between a rural and urban setting in Thailand. Findings are intended to guide Thailand Ministry of Health educational campaigns targeting arthropod-borne disease.

**Methods:**

A mixed method design was employed using a forced choice and open-ended questionnaire to assess KAP of participants seeking point-of-care treatment for malaria and/or dengue fever at government health-care facilities. Following informed consent, household construction characterization (percent eave gap, floor, wall, and roof material) and mosquito collections both indoors (using aspiration) and outside (using traps) were conducted at a subsample of participant homes. All mosquitoes were identified to genus and anopheline and aedine samples processed for potential pathogen infection.

**Results:**

A total of 64 participants were recruited from both study sites; 62 categorized as malaria symptomology and 2 categorized as dengue across all study healthcare facilities. Significant associations between study site and household construction were indicated. Trends also identified household level practices and both occupation and household construction regarding type of mosquito control products purchased and the abundance of mosquitoes in sampled homes.

**Conclusion:**

Overall, Ministry of Health information from education campaigns regarding malaria and dengue fever strategies is reaching the intended target populations at the study sites. Participants are aware of the presence of mosquitoes and that they serve as the potential vector for transmitting malaria and dengue fever diseases. However, specific knowledge gaps were also identified in each study site that may influence exposure to infected mosquitoes. Findings from this study are intended to guide future health education campaigns in these study settings to address specific community needs.

**Electronic supplementary material:**

The online version of this article (doi:10.1186/s13071-015-0653-4) contains supplementary material, which is available to authorized users.

## Background

Vector-borne diseases, such as malaria and dengue fever, account for an estimated 17% of the global burden of infectious diseases [1]. Dengue fever, caused by a flavivirus of four dengue serotypes: DEN-1, DEN-2, DEN-3, and DEN-4, is transmitted by the *Aedes aegypti* mosquito and represents one of the world’s fastest growing vector-borne diseases [2]. Acquiring one serotype will provide lifelong immunity to that particular serotype; however infections by subsequent serotypes can increase the risk of developing severe dengue symptoms [2]. Symptoms of dengue fever range from asymptomatic to mild fever, severe headache, muscle and join pain, rash to more severe hemorrhagic manifestations [2]. Malaria, another vector-borne disease of global importance, was responsible for an estimated 627,000 deaths in 2013 alone [3]. Transmitted by the bite of specific anopheline mosquitoes, which feed predominately in the evening or nighttime, infection with any of the four *Plasmodium spp.* human parasites cause symptoms of fever, headache, and vomiting which typically appear between 10–15 days after an infective mosquito bite [3]. In the absence of a marketed vaccine for either malaria or dengue fever, vector control continues to be the primary strategy to mitigate pathogen transmission.

Thailand, an endemic setting for both malaria and dengue fever disease, has been undergoing a transition from a rural and agriculture-based economy to a more urban and industrial society over the last decade [4]. This rapid urbanization has introduced new susceptible human populations into disease settings, which in turn require vector-control programs to adjust to changes in social behavior and migration patterns from rural to urban areas which may contribute to potential dengue outbreaks [5]. Dengue fever, which has had more than a 30-fold increase in incidence across the globe, caused 78,000 cases and 80 deaths in 2012 in Thailand [6]. Although a substantial decrease over recent years due to enforced vector control efforts like indoor residual spraying (IRS) and increased availability of personal protection tools like insecticide treated bed nets (ITNs) [7], malaria caused a reported 24,897 cases and 43 deaths in Thailand in 2012; of which *Plasmodium falciparum* represents 40% of cases and *Plasmodium vivax* 60% of reported cases across the country [8]. Interventions targeting the mosquito vector, such as those incorporating mosquito species-specific behaviors, have also shown to reduce the number of malaria cases [9]. One example is introducing small larvivorous fish into intra-domestic water containers for malaria vectors like *Anopheles stephensi,* which are a known to inhabit geographical regions in Thailand [9].

Commonly found in sub-tropical and tropical regions, transmission of malaria parasites and dengue virus, and subsequently health practices to reduce risk of disease, is dependent on mosquito species abundance, underlying vector ecology, as well as socioeconomic status and environmental factors [10]. In Thailand, peak transmission seasons of both malaria and dengue fever occur from June to August and from October to November which coincides with the rainy season when elevated precipitation leads to an increase in habitable breeding sites for mosquitoes [10]. This ignites many public health facilities to begin campaigning for disease prevention by performing active case detection and/or vector control measures. Insecticide treated bed nets (ITNs) are a very common form of vector-control used in Thailand and represented approximately 12.5% of the government expenditure in 2012 for malaria control [11]; however, dengue vectors are daytime-biters which make bed nets inadequate for full protection against infective mosquitoes as generally bed nets are used within the home during the nighttime hours.

Anthropogenic degradation of the environment can also contribute to changes in mosquito biodiversity (i.e., species types present) and therefore shifts in exposure of communities to varied arthropod-borne pathogens [12]. For instance, as land areas become more urban, mosquito diversity within the environment has been found to decrease [12]. Similarly, an increase/decrease in distance from mosquito breeding habitats to human blood-sources (i.e., clearing of forests), specifically in the case of malaria vectors, may alter pathogen transmission by shifting overall vector feeding patterns, vector density, and/or longevity of the mosquito all which can lead to an increased risk of arthropod-borne disease and influence health practices at the individual and/or household level [12].

Socioeconomic variables are also known to affect vector-borne disease transmission intensity. Common migration patterns within Thailand include that from rural to urban areas, and have led to urban population projections to increase from 39% of the total population in 2007 to 47% by 2027 [13]. Urbanization provides many opportunities for suitable breeding habitat of *Aedes aegypti* due to the presence of available containers for which this species is adapted (waste, tires, water storage bins) [14]. Migration also impacts housing density within communities, which has been found to increase mosquito density and play a significant role in the risk of exposure to infected vectors in crowded urban areas. In Thailand, villages with twice as many houses per unit area have been found to have significantly more *Aedes aegypti* adults and pupae than houses in villages with fewer houses per unit area [15]. Additionally, household construction characteristics are often linked to socioeconomic status and can reflect influential determinants of risk of exposure to mosquito bites [16]. For instance, the presence of window screens are typically associated with discretionary spending of the homeowner and can serve to reduce entry of mosquitoes [16]. In Thailand, tin-roofed houses have been found to have an increased risk for *Aedes aegypti* larval infestation among rural locations, whereas thatched-roofed houses have shown negative risk factors [17].

The use of qualitative information, like that of an open-ended questionnaire, can serve as a tool to gauge health practices, knowledge, and perceptions within a community to better understand gaps in use, uptake, and acceptance of vector-control programs. This information can then be shared with public health officers to guide ‘best practices’ for modifying current interventions or creating more effective strategies to include education campaigns. A study looking at dengue fever reported an increase in knowledge which led to better preventative behavior and thus a reduced burden of dengue fever disease [18]. Even more, the source of knowledge an at-risk person acquires health information from can be influential in the results observed in disease control practices. Commonly, poor and low-income families receive their information from teachers, health care workers, television, or parents [18]. Penetrating the information system to provide better knowledge will prevent wrong beliefs and common misconceptions that traditionally pass from parent to child [18].

In addition to active case detection within communities, various qualitative measures have been incorporated into the current Thai intervention and campaign schemes for vector-borne diseases. These include the use of school-based programs which target education at children, verbal education aimed at improving knowledge, radio announcements, or pamphlets, to increase campaign awareness [18-21]. Rural and urban areas have been shown to have separate campaign strategies with varied success in each [19]. For example, community-based interventions in Thailand have in past years been targeted for rural areas, and up until 2012 had not been successfully implemented in an urban setting [19]. By gaining a better understanding of individual health behaviors and/or household protection practices, these strategies can be enhanced to address specific barriers that influence the desired behavior seen at the individual or community level and thereby enhance acceptability and/or increase usage of effective protective measures. Indeed, interventions that utilize a community participatory approach to carry out mosquito control interventions have shown greater sustainability as compared to using ‘outsiders’ non-familiar to the community in a governmental top-down approach [20]. For this reason it is vital to characterize the relationship between human and coexisting vector populations in urban versus rural settings in order to better target intervention and educational campaigns in at-risk locations.

The goal of the current study was to assess the knowledge, attitudes, and practices (KAP) of at-risk populations for malaria and/or dengue fever in relation to mosquito exposure and household mosquito control practices. Specific objectives included comparison of individual and household level health practices between a rural and urban setting in Thailand. Findings are intended to guide Thailand Ministry of Health educational campaigns targeting arthropod-borne disease.

## Methods

Ethical approval was granted by The Institutional Review Boards at The University of Notre Dame, USA and Kasetsart University with informed consent of study participants conducted accordingly (Review Number:14-03-1630). A total of 18 days were spent in each study site rotated in two 9-day blocks. This schema allowed five days for conducting hospital surveys and approximately five days to perform both household mosquito collections and household construction surveys during a single site visit.

### Study sites and catchment

**Figure 1:**
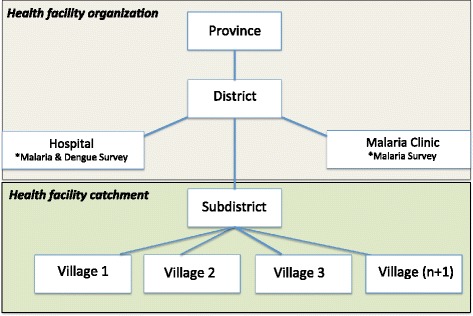
**Overview of study healthcare facility organization within provincial divisions of Thailand.** *Type of survey researchers performed at the healthcare facility.

Two political districts, Pong Nam Ron and Phanom, were selected as study sites based on endemicity for malaria and/or dengue fever and working relationships between the Ministry of Health and study personnel. Sites were characterized as either rural (Pong Nam Ron District) or urban (Phanom District) according to land use, population density, and average monthly income based on 2011 Thai government data and criteria outlined by The Royal Institute, Thailand [21-23]. Two healthcare facilities within each of the study districts were selected to serve as base field stations and location for participant recruitment. To capture both target populations; one district level hospital and one district level malaria clinic were selected. Hospital facilities were aimed at recruiting both malaria and dengue fever participants while malaria participants were captured at malaria clinic facilities. The catchment area of facilities reflected incoming populations at the sub-district and village level (Figure 1).

Pong Nam Ron District, characterized as the rural study site, is located 191 miles east of Bangkok in northwest Chanthaburi Province. Chanthaburi Province is mostly comprised of mountains, high plains, and includes several large rivers, waterfalls and natural wildlife preserves. Pong Nam Ron has a population of 42,587 people, a density of 67.17 people per mi^2^ and covers over 350 mi^2^, lying along the Cambodian border, it is divided into 5 sub-districts and 47 villages [23]. The main occupation is agricultural farming including durian, longan, potato, and corn. In 2011, the average monthly income per household was 24,278 baht (USD 755.15) [23]. Currently, there is 1 public hospital in Pong Nam Ron District and one health promoting hospital located in each of the 5 subdistricts within Pong Nam Ron District. In 2013, Pong Nam Ron had 61 cases of malaria and 5 cases of dengue hemorrhagic fever [24].

Phanom District, characterized as the urban study site, is located 441.5 miles south of Bangkok in Southern Thailand within Surat Thani Province. Phanom has a total population size of 37,950 people, a density of 85.09 people per mi^2^ and over 4977 mi^2^ of land [25]. Largely made up of dense forests and high mountain ranges; major industries within this district include rubber plantation, palm oil, coffee and fruit farms. Phanom is further divided into 6 sub-districts comprised of 56 villages [25]. In 2011 the average monthly income per household in Surat Thani was 34,417 baht (USD 1070.51) [25]. Currently, Phanom Province has 11 public hospitals in total. There is 1 hospital located in Phanom District and 9 health promoting hospitals located within 9 sub districts of Phanom District. In 2013, Phanom had 418 cases of malaria, 62 cases during the months of May and June, and 38 cases of dengue fever, 10 cases in May and 6 in June-making Phanom one of the top endemic districts within Thailand [24].

### Recruitment

Participants were recruited from study healthcare facilities using passive case detection of persons seeking point-of-care treatment for symptoms related to malaria or dengue fever. Inclusion criteria consisted of male and female adults aged 18 years and older who presented with non-life-threatening symptoms consistent with malaria and/or dengue fever, and did not require emergency medical attention. A positive diagnosis was irrelevant to participant eligibility, allowing all persons who consented to participate. Local Thai technical staff participated in recruiting eligible persons for study enrollment. A verbal explanation of the study protocol was provided in local Thai dialect, translated from English, which explained the study goals and experimental procedures. A request to survey participants’ homes for the purpose of house characterization and to collect mosquitoes was explained at the time of recruitment. One consent form was used for the KAP survey, household construction characterization survey, and mosquito collections.

### Hospital questionnaire

Convenience sampling was used to recruit eligible participants at designated study healthcare facilities. One healthcare facility was visited each day for approximately 5–7 hours over a 5 day period starting at varying times (i.e., morning, midday, and afternoon) which controlled for gender and/or age-specific biases that might occur as the result of when health care is sought (i.e. outside school, when market is closed, and/or field-work). Consenting subjects participated in a questionnaire pertaining to their KAP on malaria or dengue fever, depending on reasoning for clinic visit. Questions focused on participant’s demographic information, history of clinic attendance, individual knowledge regarding prevention of malaria and/or dengue fever disease, and individual household mosquito protection behaviors. Questions were designed based on previous KAP surveys in Thailand regarding malaria and/or dengue fever disease and were reviewed for completeness [26,27]. Each survey consisted of approximately 35 questions.

### Mosquito collections

Permission to access homes to conduct a household construction survey and to perform mosquito collections was obtained from those participants consenting to enroll in the KAP questionnaire. Household mosquito collections were performed both inside and within the immediate peri-domestic area of consenting households. Participants were provided advance notice before the house visit was made. Homes were not sampled if the head of household was absent, unless permission from a primary adult resident was granted as per informed consent during recruitment. Healthcare clinic personnel accompanied Thai technical staff and the primary research investigator to all sampled homes to facilitate collections.

Indoor mosquito collections were performed by Prokopack aspiration tool which has been proven to collect different mosquito species under different entomological conditions [28]. Sampling was performed both in the morning and evening hours to maximize probability to capture vectors based on expected resting behaviors exhibited from *Aedes* spp. and *Anopheles* spp. Prokopack collections were conducted for up to a total of 30 minutes or approximately 3 minutes per 9 m^2^. Collections were made from all rooms, if permitted, and occurred between 6:00–9:30 AM and 4:30–8:30 PM. One of two outdoor mosquito trapping methods was used depending on symptomology of the study participant. An unbaited® BG-Sentinel (Biogents AG, Regensburg, Germany) mosquito trap (BGS) was used to maximize outdoor collection of dengue vectors and an un-baited Black-hole UV light trap was used to maximize capture of outdoor malaria vectors. A single BGS trap was placed outdoors in close proximity to the entrance of the home where individuals were commonly aggregating and was operated continuously between 6:00 AM −6:00 PM. UV light traps were hung from roofs generally within 5 meters from the entrance of the home and operated continuously between 7:00 PM – 7:00 AM. Captured mosquitoes from all collection methods were held in individually labeled cartons by house code then killed on-site for identification to *Aedes* spp., *Culex* spp., *Anopheles* spp., *Armigeres* spp., or *Mansonia* spp. using morphological keys [29]. Numbers were recorded on entomologic survey forms by household, collection method, indoor or outdoor location, genus, and gender.

### Household construction characterization

Household construction characteristics were surveyed at all homes where mosquito collections were performed. Targeted variables of interest included materials used for roof, wall, and floor construction. The percentage of eave gaps, defined as the opening between the wall and roof which would allow movement of insects from outside to inside the home (i.e. exposure) was also evaluated. Additionally, the presence and condition of window screens and total amount of windows available was also recorded.

### Mosquito processing

Mosquito processing for malaria parasite or dengue virus infection status occurred at Kasetsart University in Bangkok, Thailand. A nested PCR amplification process was conducted for the detection of each *Plasmodium falciparum* and *Plasmodium vivax* within all captured female *Anopheles* spp. mosquitoes using previously established protocols [30]. The DEN-K050VectorTest® Dengue Antigen Assay (Thousand Oaks, CA) was used for assessing dengue virus infection in all female *Aedes spp.* samples following manufacturer’s specifications [31].

### Data analysis

Data was digitally entered into a master key using Microsoft Excel software and transferred to SPSS software package (SPSS version 21, Chicago, IL). Trends and patterns associated with KAP survey responses, mosquito abundance, genus type, and household construction variables were evaluated in each Phanom and Pong Nam Ron study locations. Sample data was explored for normality using box plot and histogram outputs. Statistical associations including geometric mean and median reporting were based on the deviation of sample data from a normal distribution and were performed with analyses not sensitive to normality assumptions and small sample size. Categorical data was analyzed using a Chi-Square Test of Independence or Fisher’s Exact Test. A Mann–Whitney Test was used to compare continuous variables verses categorical variables. Statistical outcomes with a p-value of 0.05 were considered significant.

## Results

### Demographics

In total, 64 persons who presented at the healthcare facilities with either malaria or dengue fever symptoms participated in the study between May-June 2014 (Table 1). Eleven participants were from Pong Nam Ron, all of whom displayed symptomology relating to malaria and 53 were from Phanom, 51 of whom displayed symptomology relating to malaria and two related to dengue fever. Seventy-three (8/11) percent of the participants in Pong Nam Ron were male, had a median age of 32 (IQR = 21–50), and identified as having a primary school education. Fifty-eight percent (31/53) of the participants in Phanom were male, had a median age of 39 (IQR = 32–47) and half identified as having up to a secondary education. One participant was unable to select his education. Almost all of the participants (73%; 7/11) within Pong Nam Ron had a farming occupation. Similarly in Phanom participants’ primary occupation was farming (62%; 33/53), followed by government officer (13%; 7/53) and other (11%; 6/53). The two participants seeking dengue fever diagnoses were aged 18 and 53, both having a secondary education and an occupation as either a government officer or other, respectively. Both study sites had a median of 4 persons living within their household (IQR = 3–5) and over 50% (Pong Nam Ron: 6/11 and Phanom: 33/53) of the respondents had no history of an infected case within their home. Occupation was the only variable significantly different between the study sites, however interpretation should be cautioned as the small sample size led to a sensitive test and significance may not be due to actual association (χ^2^ = 11.38, df = 5, N = 64, p < 0.05).

**Table 1:** **Demographic information of study participants from Phanom (urban) and Pong Nam Ron (rural) study Districts in Thailand**

**Participant characteristic**	**Phanom (urban)**	**Pong Nam Ron (rural)**	**Total**	**P-value**
	**n (%)**	**n (%)**	**n (%)**	
Total	53 (83)	11 (17)	64 (100)	
Disease				
Malaria	51 (96)	11 (100)	62 (97)	
Dengue	2 (4)	0 (0)	2 (3)	
Gender				0.505
Male	31 (58)	8 (73)	39 (60)	
Female	22 (41)	3 (27)	25 (39)	
Age				0.627
18-29	11 (21)	5 (45)	16 (25)	
30-45	26 (49)	3 (26)	29 (45)	
46-60	13 (25)	3 (27)	16 (25)	
>61	3 (5)	0	3 (5)	
Education				0.168
None	1 (2)	1 (9)	2 (3)	
Primary	21 (40)	8 (73)	29 (46)	
Secondary	26 (50)	2 (18)	28 (44)	
Bachelor	3 (6)	0 (0)	3 (5)	
Missing	1 (2)	0 (0)	1 (2)	
Occupation				0.044*
Farmer	33 (62)	7 (64)	40 (62)	
Business	1 (2)	0 (0)	1 (2)	
Employer	3 (6)	4 (36)	7 (11)	
Government officer	7 (13)	0 (0)	7 (11)	
Unemployed	3 (6)	0 (0)	3 (5)	
Other	6 (11)	0 (0)	6 (9)	

*Significant p-value.

Over half the participants (64%; 7/11) in Pong Nam Rom had never been to the selected healthcare facility before the study visit; however more than half (66%; 34/53) of the participants in Phanom had been to the selected healthcare facility for related testing, most often between 1–2 times prior. When asked why the participant visited the clinic, most from Pong Nam Ron responded that a friend, family member, or employer had recommended it. Participants from Phanom traveled to their designated facility based on close proximity to the house or for “other” reasoning. One participant seeking malaria diagnoses at the Phanom facility reported having received a negative result at the nearby malaria clinic and came to the hospital for diagnostic verification. Additional statements included that the Phanom hospital was able to test for more diseases as compared to the malaria clinic. In both locations, over 70% (49/64) of participants traveled to the clinic by driving.

### Knowledge, attitudes, and practices relating to malaria and dengue fever

Results indicated a significant difference between study sites and the frequency of participants having heard of malaria and/or dengue fever prior to their visit to the facility (p = 0.002). Sixty three percent (7/11) of participants within Pong Nam Ron confirmed to having heard of malaria or dengue fever before their visit in comparison to 98% (52/53) in Phanom. Over half (58%; 31/53) of participants from Phanom had suffered from malaria at a prior time and one out of the two dengue fever participants had suffered from dengue fever previously. In contrast, about a quarter (27%; 3/11) of the respondents in Pong Nam Ron had suffered from malaria at least once prior to their visit (Table 2).

**Table 2:** **Knowledge of vector-borne diseases from participants in Phanom (urban) and Pong Nam Ron (rural) study Districts in Thailand**

**Survey question**	**Phanom (urban)**	**Pong Nam Ron (rural)**	**P-value**
	**n (%)**	**n (%)**	
“Have you heard of malaria or dengue fever?”			0.002*
Yes	52 (98)	7 (64)	
No	1 (2)	4 (36)	
“Have you suffered from M/D before?”			0.096
Yes	31 (58)	3 (27)	
No	22 (42)	8 (73)	
“Do you know how you can get malaria and/or dengue fever?”**			
Contaminated food	2	0	1.0
Bite from a mosquito	51	9	0.410
Another person	3	1	0.508
Dirty water	1	2	0.023*

*Significant p-value.

**Forced choice question; multiple responses recorded for each respondent.

When participants in Pong Nam Ron were asked to identify how they acquired malaria or dengue fever, 82% (9/11) of the responders correctly identified ‘mosquito’ as the vector of transmission. Responses of ‘dirty water’ and ‘another person’ were also selected as causes of malaria and dengue at this study site. Asked the same question, 96% (51/53) of the participants in Phanom correctly identified the ‘mosquito’ as the vector. Responses of ‘contaminated food’, ‘dirty water’, and ‘another person’ were also selected as causes either separately or in conjunction with the belief of the mosquito vector (Table 2); of which the belief in ‘dirty water’ was the only variable of significant difference between study sites (p = 0.023). Further exploration revealed that some participants from both study sites who identified mosquitoes as the vector for malaria or dengue fever, could also state the genus. Twelve participants reported aedine mosquitoes as the vector responsible for causing malaria disease, seven participants correctly named anopheline mosquitoes as the vector for malaria, and five could match both aedine and anopheline to the appropriate disease. The KAP survey also revealed a significant difference in the source of information from whom participants’ received their knowledge specific to malaria and/or dengue fever (χ^2^ = 24.3, df = 6, N = 64, p < 0.001). However, interpretation should be cautioned as the small sample size led to a sensitive test and significance may not be due to actual association. In Pong Nam Ron, family members were the main source of malaria and dengue fever information whereas over half of the participants in Phanom reported a government official or village health volunteer as the main source of information (Table 3).

**Table 3:** **Knowledge, attitudes, and practices from participants in Phanom (urban) and Pong Nam Ron (rural) study Districts in Thailand**

**Survey question**	**Phanom (urban)**	**Pong Nam Ron (rural)**	**P-value**
	**n (%)**	**n (%)**	
Who do you get your information about malaria and dengue from?			<0.001*
None	1 (2)	4 (36)	
Television	4 (8)	0 (0)	
School teacher	1 (2)	0 (0)	
Government official	31 (58)	2 (18)	
Family member	5 (9)	4 (37)	
Other/More than one	11 (21)	1 (9)	
“I am protected from malaria if I sleep under an insecticide treated bed net”			0.933
True	46 (87)	10 (91)	
False	5 (9)	1 (9)	
Don’t Know	1 (2)	0 (0)	
Missing	1 (2)	0 (0)	
“I am only at risk of getting bit by a mosquito at night”			0.505
True	30 (56)	8 (73)	
False	21 (40)	3 (27)	
Missing	2 (4)	0 (0)	
“How often do you sleep under a bed net?”			0.220
Every night	32 (60)	8 (73)	
A few times a week	4 (8)	2 (18)	
Rarely	17 (32)	1 (9)	

*Significant p-value.

When participants were assessed on their attitude and knowledge of protection from malaria by sleeping under an insecticide treated bed net, all but one participant in Pong Nam Ron answered ‘true’. Similarly, 87% (46/53) of participants in Phanom also believed the statement was true. Two participants were unable to definitively answer this question. A response from one participant in Phanom stated that while the statement is true “*it only protects you from malaria 90% of the time*”. About three-quarters of participants (73%; 8/11) in Pong Nam Ron perceived their only risk to getting a mosquito bite was during the night. In Phanom, 57% (30/53) believed this to be true and one participant even stated “*there are no infective mosquitoes during the day time*”. Some participants within Phanom perceived most of their risk to occur at night, but only sometimes during the day. These participants ultimately answered ‘false’ to ‘sleeping under an insecticide treated bed net will protect me from malaria’ (Table 3). Overall, there were no significant differences indicated in both knowledge of bed net use (p = 0.933) and attitudes towards risk from mosquito bites (p = 0.505) between study sites. Additionally, no significant difference existed with regard to the frequency participants reported sleeping under a bed net (p = 0.220). When participants in Pong Nam Ron were asked how often they sleep under a bed net, 73% (8/11) responded with ‘every night’, followed by a ‘few times a week’ (18%; 2/11) and ‘rarely’ (9%; 1/11). When asked the same question in Phanom, 60% (32/53) responded with ‘every night’ and 32% (17/53) responded ‘rarely’ (Table 3). At both study sites, 10% of participants reported owning 0 bed nets, with a median of 2 bed nets owned by each participant. As one participant stated, “*I do not believe in sleeping under an impregnated bed net, if your hand touches the bed net you can still get bit*”.

When participants were asked to list prevention methods they knew would protect them against diseases caused by mosquitoes, bed nets and repellents were the most common responses from participants in Pong Nam Ron, followed by wearing long sleeves, pants and burning coils. Other prevention methods mentioned included using fire or smoke, a fan, treating containers with temephos (a larvacide), and fish. Participants in Phanom also commonly responded with bed nets and repellents as methods to prevent diseases caused by mosquitoes as well as destroying containers, temephos, and coils followed by fire or smoke, long pants and sleeves, IRS or fogging, a fan, bug zapper, fish, eating healthy, and exercise. One urban participant reported that placing salt water in cups underneath table legs was an appropriate control method (data not shown).

In Pong Nam Ron, when asked how the participant could tell if another person was sick with malaria, 73% (8/11) of patients stated they didn’t know and only 27% (3/11) were able to correctly mention one or more correct symptoms, with headache and fever being most frequently mentioned. When participants in Phanom were asked the same question, 82% (42/51) were able to correctly name one or more corresponding symptoms with headache, fever, and chills most commonly mentioned. Additionally, both dengue fever participants were able to correctly identify at least one symptom related to dengue, mentioning a high fever, headache and rash; with one of the dengue fever participants stating that malaria and dengue fever had the same symptoms, mentioning headache, cyclic fever, and chills.

### Household mosquito control practices

When participants were asked about protective measures they use in their home to control mosquitoes, 54% (6/11) of participants within Pong Nam Ron and 90% (45/50) in Phanom responded that they paid for a personal protective tool outside of what might have been given to them by the Thai MOH and/or other organizations. Of the products purchased, repellent creams and coils were the most frequently named products in both Pong Nam Ron and Phanom, although in Phanom, bed nets were also included in the most frequently named products (Additional file 1). However, one participant in Phanom stated, “*The officer gave me a bed net, but I do not use because it is too small so I bought a new one*”. Another participant declared, “*I don’t like the bed net with chemical, I bought one without chemicals*”. The majority (81%; 52/64) of participants within both study sites considered mosquitoes to be a problem at their house and 90% (58/64) reported seeing mosquitoes inside their home every day. When asked whether the participant would buy additional products to protect themselves out of fear of disease or to protect against annoying mosquito bites, the overwhelming response in both study sites was to prevent mosquito bites (Pong Nam Ron: 8/9 and Phanom: 38/45).

Overall, participants from both study sites reported having personnel come out to their household to discuss ways to prevent malaria and/or dengue fever. Participants from Pong Nam Ron reported government officers as the most common personnel to visit and in Phanom both government officers and voluntary health workers were regularly mentioned. Reported activities performed by these personnel included: IRS spraying, verbal education, providing bed nets, temephos treatment of containers, and performing blood testing.

### Household mosquito collections

In total, 53 participants gave consent to study personnel for household construction characterization and mosquito collection. Of those, a total of 34 households were selected; 8 in Pong Nam Ron (rural) and 26 in Phanom (urban). Final sample size was a result of both logistical feasibility and accessibility to enter homes at time of survey. All 8 homes characterized in Pong Nam Ron were associated with participants seeking malaria treatment. In Phanom, 25 homes were from participants seeking malaria diagnoses and 1 participant for dengue fever. A total of 149 *Anopheles* spp., 209 *Aedes* spp., 438 *Culex* spp., 38 *Armigeres* spp., and 7 *Mansonia* spp. were captured from all trapping methods throughout the study period. The total number of mosquitoes captured indoors by prokopack across both study sites was 501 (Pong Nam Ron: 162 and Phanom: 339) from a total of 6 and 24 homes, respectively. The total number of mosquitoes collected by outdoor UV light trap was 414 across both sites (Pong Nam Ron: 44 and Phanom: 370) from a total of 6 and 24 homes, respectively. Seven mosquitoes were captured by outdoor BG trap from the 1 home sampled in Phanom.

Overall, the total number of mosquitoes collected inside the homes at Pong Nam Ron was higher than outside while collections made in Phanom was highest outside surveyed homes (Additional file 2). The geometric mean number of mosquitoes captured indoors in homes within Pong Nam Ron was 6.8 and outside the home was 3.7. In Phanom, the geometric mean number of mosquitoes collected indoors was 5.43 and outside the home was 10.75. At both study sites, *Culex* spp. was the dominant mosquito genus collected from outdoor traps as well as within homes at Pong Nam Ron. Over 50% of the collections performed within homes at Phanom were *Aedes* spp. The majority (14%) of anophelines captured outside in Phanom were from the UV light trap whereas in Pong Nam Ron (rural) equal proportions of anophelines (27%) were collected using Prokopack aspiration (inside) and the UV light trap (outdoors). A significant difference was indicated in the total number of mosquitoes found outside the home between study sites (*U* = 134, *n*_1_ = 7, *n*_2_ = 25, p < 0.05). However, the total number of mosquitoes found within the home was not (p = 0.560).

### Household characterization

Of the 8 homes surveyed in the rural site of Pong Nam Ron, the majority were constructed with a tin roof, a wood floor, wood or mixed walls, and 50% had an eave gap of >75%. On the contrary, the majority of surveyed homes in Phanom had a concrete roof, tile floor, cement walls, and an eave gap < 25% (Table 4). Results indicated a significant difference in roof (χ^2^ = 25.8df = 3, N = 34, p < 0.001), wall (χ^2^ = 21.2, df = 3, N = 34, p < 0.001), floor construction (χ^2^ = 23.6, df = 3, N = 34, p < 0.001), and eave gap (χ^2^ = 15.23, df = 3, N = 34, p = 0.002) between the two study sites; however interpretation should be cautioned as the small sample size led to a sensitive test and significance may not be due to actual association.

**Table 4:** **Frequency of household construction characteristics of study participant homes in Phanom (urban) and Pong Nam Ron (rural) study Districts within Thailand**

	**Phanom**	**Pong Nam Ron**	**Total**	**P-value**
	**n (%)**	**n (%)**	**n (%)**	
Total	26 (76)	8 (23)	34 (100)	
Roof construction				<0.001*
Concrete/Masonry	24 (92)	1 (13)	25 (74)	
Tin	0 (0)	6 (75)	6 (17)	
Mixed	1 (4)	1 (12)	2 (6)	
Other	1 (4)	0 (0)	1 (3)	
Floor construction				<0.001*
Concrete	5 (20)	3 (38)	8 (23)	
Tile	21 (80)	0 (0)	21 (62)	
Dirt	0 (0)	1 (12)	1 (3)	
Other	0 (0)	4 (50)	4 (12)	
Wall construction				<0.001*
Cement	24 (92)	1 (12.5)	25 (74)	
Bamboo	0 (0)	1 (12.5)	1 (3)	
Wood	0 (0)	2 (25)	2 (6)	
Mixed	1 (4)	2 (25)	3 (8)	
Other	1 (4)	2 (25)	3 (9)	
% of Eaves open				0.002*
Less than 25%	14 (54)	3 (38)	17 (50)	
25-50%	10 (38)	1 (12)	11 (32)	
50-75%	2 (8)	0 (0)	2 (6)	
More than 75%	0 (0)	4 (50)	4 (12)	

*Significant p-value.

### Mosquito collections, household eave gap and relation to mosquito control practices

**Figure 2:**
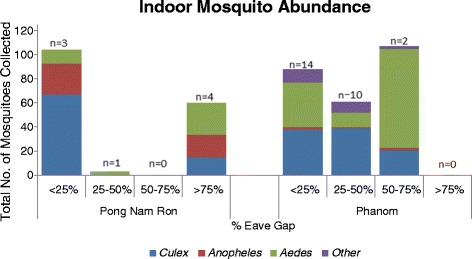
**Indoor mosquito abundance at participant households by eave gap classification (n = homes sampled).**

In Pong Nam Ron mosquitoes were collected from participant homes that represented eave gap classifications of <25%, 25-50%, and >75%. Homes with < 25% eave gap had an overall higher abundance of mosquitoes inside homes from Pong Nam Ron in comparison to Phanom (Figure 2). Homes in Pong Nam Ron that had an eave gap > 75% had a geometric mean of 5.8 mosquitoes collected from indoors in comparison to 8.4 mosquitoes which was the geometric mean for homes <25%. Alternatively, households within Phanom represented eave gap classifications of <25%, 25-50%, and 50-75%. Homes with an eave gap of 50-75% had a geoemetric mean of 26.5 mosquitoes collected indoors in comparison to 4.6 mosquitoes collected from homes <25% eave gap (Additional file 3).

**Figure 3:**
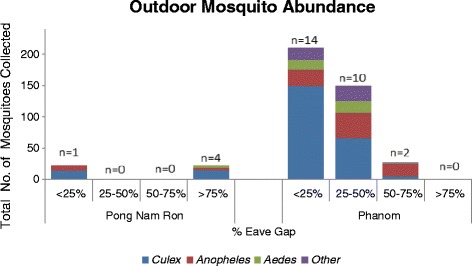
**Outdoor mosquito abundance at participant households by eave gap classification (n = homes sampled).**

Specifically, *Culex* spp. was collected in greatest abundance inside homes with < 25% eave gap in Pong Nam Ron (rural) whereas *Aedes* spp. was found in greater abundance within homes with similar eave gap classifications in Phanom (urban). The abundance of *Anopheles* spp. was greatest in Pong Nam Ron as compared to collections in Phanom with similar eave gap openings (Figure 2). The greatest abundance of outdoor mosquitoes represented *Culex* spp. in both study sites (Figure 3). In Phanom, homes with < 25% eave gap had higher outdoor trap collections as compared to homes with a greater eave gap. *Aedes* spp. were found in greater abundance outside of homes within Phanom as compared to Pong Nam Ron. *Anopheles* spp. were trapped in higher abundance from homes in Phanom verses Pong Nam Ron; although not influenced by eave gap characterization (Figure 3).

**Figure 4:**
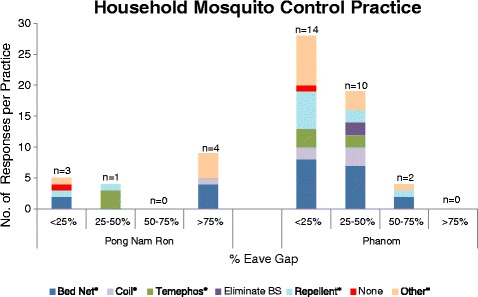
**Mosquito control products utilized at participant households by eave gap classification (n = homes surveyed).** *Product mentioned was purchased; “other” refers to bug zappers and fans.

Among participants from Pong Nam Ron, bed nets, temephos, repellents, and other household products were reportedly used for household protection from malaria and/or dengue fever. Participants in Phanom mentioned bed nets, coils, repellents, and eliminating mosquito breeding sites. Participants from homes within Pong Nam Ron that had >75% eave gap reported having used more products in general than homes with a 0-50% eave gap (Figure 4). In Phanom, participants reported purchasing more products overall as compared to the rural study site. Repellent and coil usage were methods reported more often from participants whose homes had a 0-50% eave gap verses homes with an eave gap > 50%. In both study sites, participants that reported having no household control methods were from houses with <25% eave gap (Figure 4). In addition, as homes were characterized with less of an eave gap (0-50%), there was a matched participant survey response of lack of bed net use; 100% of participants residing within homes that had an eave gap of >75% responded with the use of a bed net (Additional file 4).

### Mosquito infection processing

A total of 84 female anophelines out of the149 collected by both Prokopack and UV light trap methods were processed for the detection of *P. falciparum* and *P. vivax* infection status. Of those tested, 29 were confirmed negative: 3 from collections in Pong Nam Ron and 26 from collections in Phanom. The remaining 55 *Anopheles* spp. samples were collected from Phanom and indicated a false positive infection by malaria nested PCR protocol. These samples will be retested under a separate nested PCR protocol using alternative primers.

The sampled household from the dengue fever participant resulted in zero female *Aedes spp*. mosquitoes and thus RDT dengue detection was not applicable.

## Discussion

Key determinants of successful vector control programs include implementation of site-specific strategies that strengthen community buy-in required for long-term sustainability [10]. The current study employed a mixed method approach to assess relationships among household vector control activities, housing construction characteristics, and individual KAP in relation to malaria and/or dengue fever. Overall, while statistical evidence should be interpreted with caution, results revealed important trends between the two study sites, one urban and one rural, in Thailand that may directly impact household disease prevention practices.

Participants’ perceptions of risk of acquiring malaria and dengue fever were similar within both sites. Over 75% of the participants in Pong Nam Ron and 90% of those in Phanom were correctly able to identify the mosquito as the primary vector responsible for transmitting malaria and dengue fever. However, participants’ were unable to name the genus of the mosquito vector (*Anopheles or Aedes)*, or incorrectly identified the mosquito genus associated with a particular disease etiology despite government officers and healthcare personnel from study healthcare facilities confirming that educational campaigns provide the proper genus name of the mosquito responsible for disease transmission. This confusion with regard to mosquito identification highlights the potential gap in absorbing information presented during educational campaigns and how this may directly translate to inappropriate actions used to protect themselves from malaria and dengue fever in their home. The inability to recall that malaria and dengue diseases are transmitted by two different mosquitoes which exhibit different behaviors and biology, most importantly that *Aedes aegypti* dengue vectors bite during the day and *Anopheles spp.* malaria vectors bite predominately at night, plays a crucial role in the personal protective behaviors practiced by the participant. Without this basic understanding, a participant’s perception of risk and how best to prevent exposure to these pathogens may be compromised. This may be what underlies the shared belief from more than half of participants that mosquitoes are only capable of biting in the evening, with one participant even stating, “*there are no infective mosquitoes during the day time*”.

Trends in participants’ knowledge related to prevention methods for mitigating malaria and dengue fever similar among the two study locations. This was surprising as increasing socioeconomic status often leads to more access to education and potential access to knowledge surrounding these diseases. In most cases, more than one prevention method was mentioned by each participant, suggesting education campaigns are adequately addressing this issue. However, there were some shared knowledge gaps in understanding why these products are used as one participant explained, “*I use a coil in my home, but I do not know why*”. These knowledge gaps may be due, in part, to current practices of community education. Specifically, malaria clinics are primarily responsible for implementing intervention campaigns for malaria and dengue fever campaigns and household intervention methods, such as distribution of temephos and elimination of breeding sites, are the responsibility of separate healthcare facilities. Both approaches incorporate home visits, resulting in residents being exposed to intervention and campaign work for both diseases which may create an overabundance of information thereby making it difficult for residents to distinguish appropriate household preventive measures for each malaria and dengue fever. The result is a potential false sense of practiced protection. Motivation for practicing mosquito control methods in both study sites was predominately due to annoyance from mosquito bites versus a concern for disease transmission. This suggests that no relationship yet exists between the understanding that even one mosquito is enough to cause a health concern and transmit disease. Even after performing mosquito collections at participants’ homes, such expressions like “*that is not too many*” highlights the continued need for health education.

Nearly every participant considered mosquitoes to be a problem at their home and purchased products for household mosquito control outside out of what was given to them for free. The fact that over half of the participants from Phanom reported having suffered from malaria at a prior time may have contributed to the numbers and types of products purchased. The choice of products purchased between study sites showed trends related to occupation. Participants from Phanom (urban) were most often associated with farming occupation, particularly rubber plantation farming, which predisposes participants being exposed to anopheline vectors during the nighttime hours because this is when latex is harvested. Results revealed not only a higher abundance of mosquitoes found outside the home in comparison to indoors, but specifically a high abundance of *Anopheles* spp. Related occupations from this area may have led to participants’ to have perceived mosquito annoyance outside the home and therefore influenced products and behaviors practiced such as using topical repellents and temephos.

In comparison, participants from Pong Nam Ron (rural), where agriculture farming particularly fruit orchard farming, identified themselves as an employee or farmer. Such occupations predispose participants to daytime biting mosquitoes when fruit it typically harvested. Indeed, overall mosquito abundance and anophelines in particular, was greater indoors in comparison to outside the home. Participants from Pong Nam Ron not only reported a more frequent practice of sleeping under a bed net every night, but also mentioned the purchase of coils and fans that are used inside the home. This difference in occupation, and/or associated discretionary spending, may have influenced motivation for products purchased and household practices performed. Lastly, while the majority of participants stated they practiced personal protection by sleeping under a bed net, a number of participants revealed that the bed net used was not the recommended ones provided by healthcare officials. Sometimes participants would purchase their own untreated nets citing negative attitudes towards chemicals or the small bed net size. Further investigation into the beliefs and perceptions of treated versus untreated bed nets is warranted and would be beneficial to better inform on safety of insecticides to humans, patterns of net distribution, re-treatment instruction as well as proper usage of nets in these target populations. Of particular interest, participants who reported ‘rarely’ sleeping under a bed net came from both study sites in housing construction with a 0-50% eave gap (Additional file 4) suggesting that as housing structure improves, participant’s individual health practices are influenced.

Household construction characteristics were also shown to be associated with mosquito density trends that may have influenced survey responses between sites. For instance, in general there was improved housing construction observed in Phanom, the urban setting, whereby concrete was used for roof and wall construction, with flooring being tiled. The homes also exhibited eaves gaps representing <25%, which may have mitigated exposure to mosquitoes inside the home. However, when comparing entomological data from homes with similar eave gap percentage between study sites, we did not see strong trends suggesting greater mosquito density within homes from Pong Nam Ron whose housing construction materials were overall poorer. What the data did reveal was that nearly twice as many mosquitoes were collected on average within homes in Pong Nam Ron as compared to outside the home. This was a stark difference as compared to Phanom where nearly twice as many mosquitoes were collected on average outside the home as compared to within. A larger sample size (i.e., community level) is necessary to further explore relationships among housing, vector exposure, and KAP.

There were several limitations of this study that should be noted. First, the study period occurred during the rainy season, which resulted in heavy and prolonged rain lasting several days. Small participant sample sizes might have resulted from difficulty traveling to healthcare facilities and/or fewer persons being able to seek medical attention and not necessarily representative of actual disease presence within the study site. Additionally, as the study design employed passive compared to active surveillance, this most likely further reduced the number of potential participants. However, based on MOH data the year prior to the study, it was anticipated that cases of both malaria and dengue fever would be presenting at study facilities during the months of May and June [24].

Potential biases in mosquito collections may have also occurred as a result of providing advance notice of when household visits would occur and/or the accompaniment of government officers to the participant homes. Both scenarios may have altered homeowner mosquito control practices prior to study personnel arrival.

Lastly, limitations occurred in the total number of mosquito collections and household characterization surveys that were able to be performed which increased the chance for bias due to low replication. Larger study teams that can be used to cover more area and greater effort to sensitize the participant on link of mosquito collections to overall program goal are recommended for future studies to increase willingness to participate and household sample size. The socioeconomic status of participants influenced the placement of UV light traps used for mosquito collections (i.e., access to electricity) that resulted in mosquito trapping being performed up to 500 meters from the home. In those instances, captured mosquitoes may not represent densities and/or species immediately outside the participant’s home and potentially encountered by the participant. Finally, there were also two households that had a fire with smoke during mosquito collections that may have decreased mosquitoes inside the homes thereby misrepresenting actual densities.

Findings from this pilot study have identified several areas where government campaigns and interventions could be targeted to enhance individual health behavior and household mosquito control practices. This includes initiating an open platform collaborating educational campaign for both malaria and dengue fever among healthcare facilities. Combining efforts simultaneously may allow for a more cohesive intervention program. A mobile device platform could be used to track activities of village health volunteers and Ministry of Health personnel from their respective campaigns. Data tracking the home visited, intervention actions performed, education discussed, and/or any positive cases reported by active surveillance could be collected. This mobile platform could be monitored in real-time and be accessed by both parties to provide a more comprehensive approach of education, outbreak, or clusters of positive cases in addition to filling any gaps in intervention strategies or education needed to tackle both vector-borne diseases. One possible intervention action to be performed during household visits by healthcare workers may be to incorporate occupation linked vector control strategies. Occupation-based interventions, such as DEET impregnated soap, insecticide treated hammocks and personal clothing, have been shown to reduce the number of malaria cases in countries such as Pakistan, Afghanistan, and Vietnam [32]. Similar approaches may prove useful within the current study communities based on trends associating occupation to the type of vector control products purchased by homeowners identified in our surveys.

Another collaborative approach could reflect a “Village Health Day” where available medical services are held within the community. Opportunities to increase access to mosquito control tools such as repellent or coils could be distributed through such organized events. Sample data suggests that participants from urban areas purchased more mosquito control vector tools in comparison to participants from the rural setting. Subsidizing these products during select campaigns would benefit communities with less access to these products, and provide an opportunity for direct outreach and information exchange. Either of these community-level approaches could greatly benefit resource-limited facilities as well as streamline vector-borne disease approaches for better up-take. Finally, increasing capacity to detect multiple vector-borne diseases at a single healthcare facility, or increasing the number of point of care treatment access sites would greatly benefit community members. Convenience to clinics factored into participants’ treatment seeking behavior. Increasing the number of clinics available for diagnostic testing may encourage more frequent facility visits and therefore reduce probability of disease outbreaks due to human reservoirs. A specific example would be the introduction of dengue RDT kits to malaria clinics.

## Conclusion

Overall, education from malaria and dengue fever intervention campaigns is reaching the intended target populations. Target populations are aware of the presence of mosquitoes and that they serve as the potential vector for transmitting malaria and dengue fever diseases. However, data from this pilot study suggests there are gaps in knowledge and perception of risk across participants in both study sites. The current pilot study serves as a platform for future longitudinal studies to assess individual perceptions and household practices related to the prevention of malaria and dengue fever. Continued monitoring of the variables measured here would be expected to facilitate an understanding of disease transmission trends as the study locations and serve as a tool to guide vector control strategies, including educational campaigns, within communities at-risk for these diseases. Combined, this would inform on best approaches to household mosquito control practices for protection against arthropod-borne disease. Further goals include improving the KAP of endemic populations, reducing misconceptions of risk, increasing overall individual health practices and understanding of preventative tools.

## Additional files

Additional file 1:
**KAP questionnaire responses from participants whose households were sampled for mosquito collections.**
Additional file 2:
**Total abundance of mosquitoes collected both inside and outdoors of participant households (n= homes sampled).**
Additional file 3:
**Geometric mean number of mosquitoes collected inside participant households by eave gap classification (n = homes sampled).**
Additional file 4:
**Reported household bed net use at participant households by eave gap classification (n= homes sampled).**

## Abbreviations

KAP: Knowledge, attitudes, and practices
MOH: Ministry of Health
IRS: Indoor residual spraying
ITN: Insecticide treated bed nets
BGS: An unbaited ®BG-Sentinel (Biogents AG, Regensburg, Germany) mosquito trap
PCR: Polymerase chain reaction
IQR: Interquartile range
RDT: Rapid detection test
