# Supplementary material for: Targeting educational campaigns for prevention of malaria and dengue fever: an assessment in Thailand
Source: Parasit Vectors. 2015 Jan 23;8:43. doi: 10.1186/s13071-015-0653-4 (PMC4311424; doi:10.1186/s13071-015-0653-4)
Supplement: Additional file 1: — KAP questionnaire responses from participants whose households were sampled for mosquito collections. [file 13071_2015_653_MOESM1_ESM.pdf]

KAP, Household Mosquito Collection, and Household Construction Consolidated Chart

Patient ID[A]	Disease Symptom[B]	Mosquito Genus[C]								Household Construction[D]				Method used at the home[E]	Occupation[F]	How often slept under BN[G]
		Culex spp.	*Anop spp.*	*Aedes spp.*	*Arm spp.*	*Man spp.*	Total			Roof	Wall	Floor	% Eave Gap			
							Inside	Outside	Grand Total				<25, 25-50, 50-75, >75			
1	M	5	0	1	0	0	2	4	6	Tin	Mixed	Other	>75	BN	Em	Every
2	M	1	0	1	0	0	1	1	2	Tin	Mixed	Other	>75	BN, C,S,L	Em	Every
3	M	7	16	27	0	0	44	6	50	Con	Cement	Con	>75	BN	Em	Few
4	M	17	7	0	0	0	13	11	24	Tin	Wood	Con	>75	BN,F	F	Few
5	M	3	2	0	0	0	3	2	5	Tin	Wood	Wood	<25	None	Em	Every
7	M	0	1	1	0	0	2	-	2	Tin	Other	Other	<25	BN, F	F	Every
8	M	78	31	10	0	0	99	20	119	Mixed	Other	Con	<25	BN,R	F	Rarely
38	M	0	0	3	0	0	3	0	3	Tin	Bamboo	Dirt	25-50	R	F	Rarely
11	M	8	4	2	3	0	10	7	17	Con	Cement	Tile	25-50	C	U	Every
13	M	8	0	5	1	2	2	14	16	Con	Cement	Tile	<25	R, BN	F	Rarely
15	M	0	0	0	0	0	0	-	0	Con	Cement	Tile	<25	None	O	Every
18	M	7	3	8	8	0	10	16	26	Con	Cement	Tile	<25	BN,F,S	G	Every
19	M	6	3	0	0	1	1	9	10	Con	Cement	Tile	<25	BN, R	F	Rarely
20	M	8	1	11	3	0	15	8	23	Con	Cement	Tile	<25	C, F	G	Every
23	M	3	3	0	1	0	1	6	7	Con	Cement	Con	25-50	BN	F	Every
24	M	19	6	2	3	1	6	25	31	Con	Cement	Con	25-50	BN,R	F	Every
26	M	10	2	5	0	0	4	13	17	Con	Cement	Con	<25	BN	F	Every
30	M	4	0	1	5	0	9	1	10	Con	Cement	Tile	<25	BN, R, S	G	Every
32	M	3	1	1	0	2	0	7	7	Con	Cement	Tile	<25	BN	F	Every
34	M	19	20	3	2	0	2	42	44	Con	Cement	Tile	25-50	BN,T, E	F	Every
35	M	21	3	81	3	0	100	8	108	Con	Cement	Con	50-75	BN, R	F	Every
36	M	12	2	6	9	0	12	17	29	Con	Cement	Tile	25-50	BN, C, F	F	Rarely
37	M	79	3	5	2	0	9	80	89	Con	Cement	Tile	<25	R, S	U	Every

41	M	4	1	3	1	0	3	6	9	Con	Cement	Tile	25-50	C,R, BN	F	Every
42	M	7	4	0	0	0	2	9	11	Con	Cement	Tile	25-50	S, BN	F	Every
44	M	7	1	0	0	1	-	9	9	-	-	-	-	BN, Fo	F	Few
45	M	5	19	2	0	0	7	19	26	Con	Cement	Tile	50-75	T,R,BN, F,L	F	Rarely
46	M	17	3	2	2	0	9	15	24	Con	Cement	Tile	<25	R, T,F	G	Every
48	M	7	3	3	0	0	4	9	13	Con	Cement	Tile	<25	BN, T, Z	O	Rarely
49	M	27	4	3	1	0	11	24	35	Con	Cement	Tile	<25	E	F	Every
50	M	15	1	4	4	0	24	-	24	Con	Cement	Tile	25-50	BN, Z	F	Every
51	M	14	0	4	9	0	12	15	27	Con	Cement	Tile	25-50	BN, C, E, L	U	Every
52	M	4	1	6	0	0	4	7	11	Mixed	Mixed	Con	25-50	BN,T	F	Every
55	M	2	3	4	2	0	7	4	11	Con	Cement	Tile	<25	C,BN,F, T,R	F	Rarely
59	D	9	0	4	1	0	7	7	14	Con	Cement	Tile	<25	E, S	O	Rarely

[A] Patient ID 1-5,7-8 and 38 were from Pong Nam Ron, all others were from Phanom
[B] Disease Symptomology: M=Malaria D=Dengue fever
[C] Mosquito Genus: *Culex* spp., *Anopheles* spp., *Aedes* spp., *Armigeres* spp., *Mansonia* spp.
[D] Household Construction: Con=concrete
[E] Method used at the home: C=coil, BN=bed net, F=fan, T=tempehos, R=repellent, E=eliminate breeding site, S=fire/smoke, L=long sleeve, Z=bug zapper, Fo= fogging
[F] Occupation: F= farmer, E=employee, G=government officer, U=unemployed, O=other
[G] How often slept under a bed net: Every = every night, Few = a few times a week, Rarely = rarely
