# Supplementary figures and images for: Targeting educational campaigns for prevention of malaria and dengue fever: an assessment in Thailand
Source: Parasit Vectors. 2015 Jan 23;8:43. doi: 10.1186/s13071-015-0653-4 (PMC4311424; doi:10.1186/s13071-015-0653-4)

## Mosquito Abundance (Total)

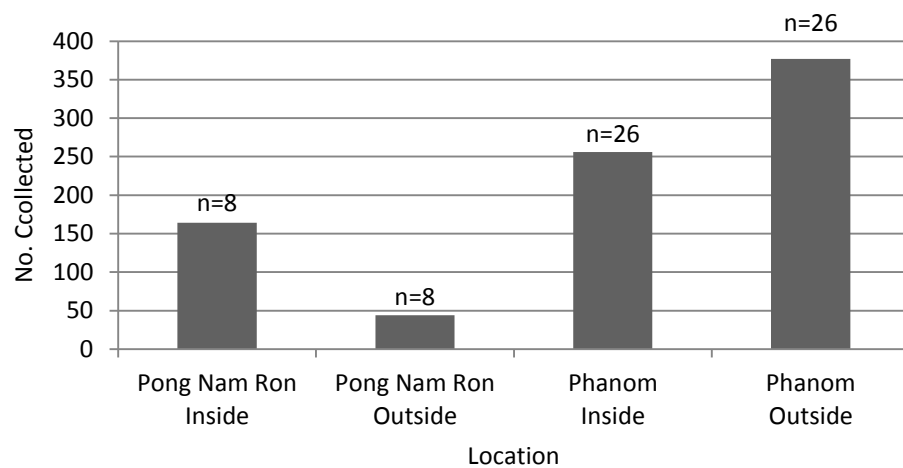

Supplement: Additional file 2: — Total abundance of mosquitoes collected both inside and outdoors of participant households (n= homes sampled). [file 13071_2015_653_MOESM2_ESM.pdf]

## Indoor Mosquito Abundance (Geometric Mean)

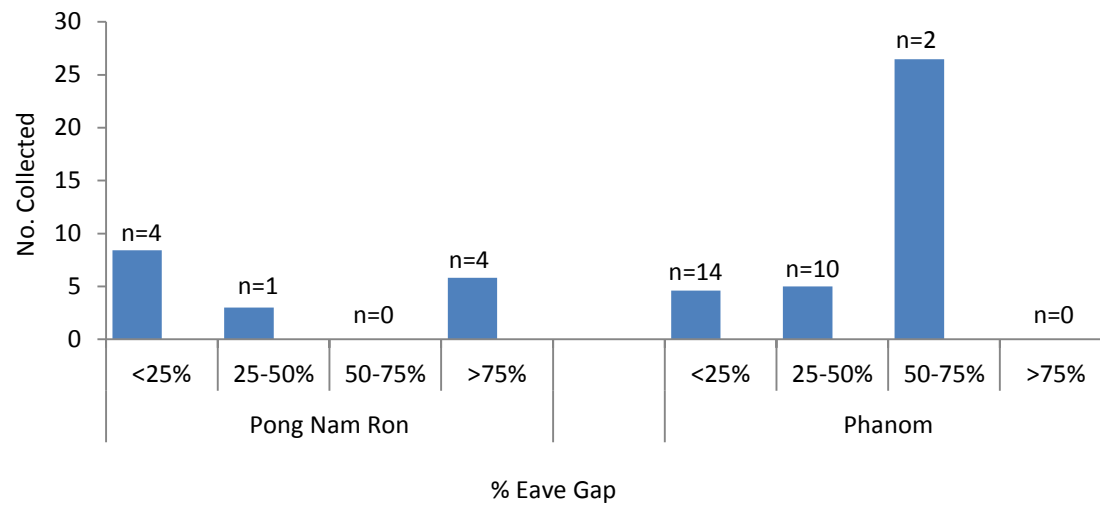

Supplement: Additional file 3: — Geometric mean number of mosquitoes collected inside participant households by eave gap classification (n = homes sampled). [file 13071_2015_653_MOESM3_ESM.pdf]

## Household Eave Gap and Bed Net Use

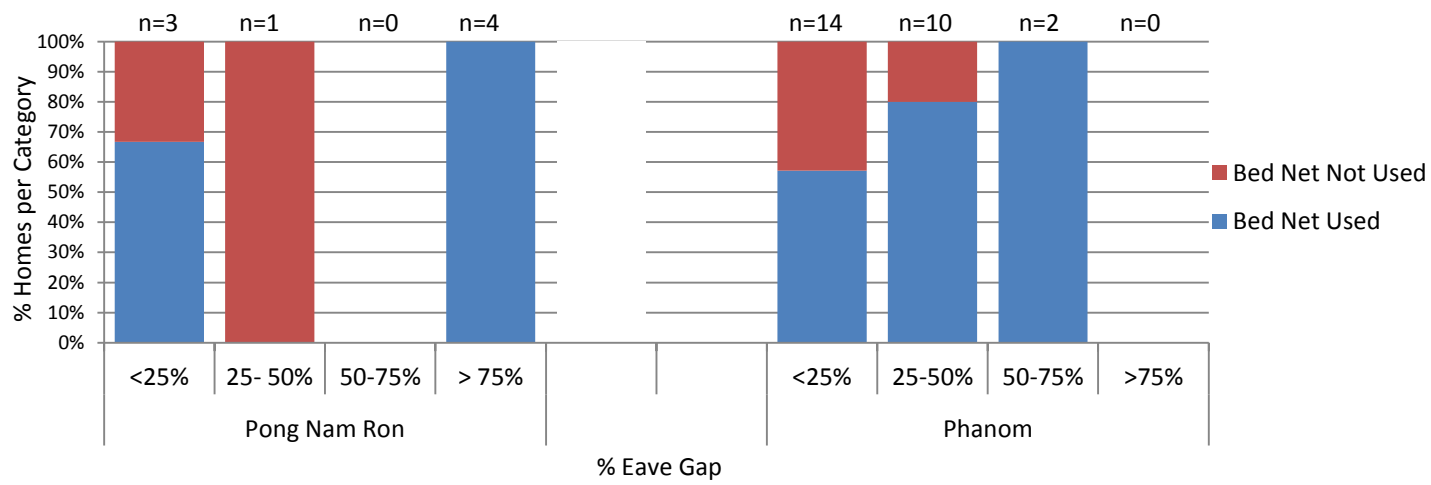

Supplement: Additional file 4: — Reported household bed net use at participant households by eave gap classification (n= homes sampled). [file 13071_2015_653_MOESM4_ESM.pdf]
